# Supplementary material for: Heparin-based hydrogel scaffolding alters the transcriptomic profile and increases the chemoresistance of MDA-MB-231 triple-negative breast cancer cells
Source: Biomater Sci. 2020 Feb 13;8(10):2786–96. doi: 10.1039/c9bm01481k (PMC7497406; doi:10.1039/c9bm01481k)
Supplement: Supplementary file 2 [file BM-008-C9BM01481K-s002.zip › Supplementary File 4/HP-BhydrogelvControl/Pathways/my_analysis.Gsea.1545201079907/HALLMARK_UV_RESPONSE_UP.html]

Details for gene set HALLMARK\_UV\_RESPONSE\_UP[GSEA]

|  || Dataset | expr.class.cls#HYD\_versus\_CONTROL.class.cls#HYD\_versus\_CONTROL\_repos |
| Phenotype | class.cls#HYD\_versus\_CONTROL\_repos |
| Upregulated in class | CONTROL |
| GeneSet | HALLMARK\_UV\_RESPONSE\_UP |
| Enrichment Score (ES) | -0.21281612 |
| Normalized Enrichment Score (NES) | -0.9720438 |
| Nominal p-value | 0.5294118 |
| FDR q-value | 0.54084235 |
| FWER p-Value | 0.999 |
Table: GSEA Results Summary

  

Fig 1: Enrichment plot: HALLMARK\_UV\_RESPONSE\_UP      
 Profile of the Running ES Score & Positions of GeneSet Members on the Rank Ordered List

  

| PROBE | DESCRIPTION (from dataset) | GENE SYMBOL | GENE\_TITLE | RANK IN GENE LIST | RANK METRIC SCORE | RUNNING ES | CORE ENRICHMENT || 1 | CLCN2 | na |  |  | 285 | 2.697 | 0.0167 | No |
| 2 | DGAT1 | na |  |  | 636 | 2.172 | 0.0239 | No |
| 3 | WIZ | na |  |  | 893 | 1.959 | 0.0335 | No |
| 4 | ONECUT1 | na |  |  | 1628 | 1.520 | 0.0128 | No |
| 5 | STK25 | na |  |  | 1846 | 1.424 | 0.0182 | No |
| 6 | SHOX2 | na |  |  | 1871 | 1.418 | 0.0335 | No |
| 7 | FOSB | na |  |  | 1957 | 1.379 | 0.0453 | No |
| 8 | STARD3 | na |  |  | 1967 | 1.377 | 0.0610 | No |
| 9 | SULT1A1 | na |  |  | 2186 | 1.280 | 0.0646 | No |
| 10 | CLTB | na |  |  | 2391 | 1.212 | 0.0681 | No |
| 11 | ATF3 | na |  |  | 2583 | 1.150 | 0.0716 | No |
| 12 | NR4A1 | na |  |  | 2699 | 1.116 | 0.0787 | No |
| 13 | CDC34 | na |  |  | 2900 | 1.063 | 0.0807 | No |
| 14 | POLG2 | na |  |  | 3014 | 1.034 | 0.0869 | No |
| 15 | SLC6A8 | na |  |  | 3116 | 1.006 | 0.0934 | No |
| 16 | TCHH | na |  |  | 3197 | 0.988 | 0.1008 | No |
| 17 | ACAA1 | na |  |  | 3257 | 0.975 | 0.1092 | No |
| 18 | CHKA | na |  |  | 3515 | 0.914 | 0.1064 | No |
| 19 | FURIN | na |  |  | 3731 | 0.863 | 0.1053 | No |
| 20 | ARRB2 | na |  |  | 3818 | 0.847 | 0.1107 | No |
| 21 | RASGRP1 | na |  |  | 3920 | 0.828 | 0.1151 | No |
| 22 | NXF1 | na |  |  | 4257 | 0.760 | 0.1064 | No |
| 23 | MGAT1 | na |  |  | 4442 | 0.725 | 0.1053 | No |
| 24 | CYP1A1 | na |  |  | 4586 | 0.695 | 0.1060 | No |
| 25 | APOM | na |  |  | 4812 | 0.657 | 0.1019 | No |
| 26 | BMP2 | na |  |  | 4891 | 0.643 | 0.1054 | No |
| 27 | DLG4 | na |  |  | 4990 | 0.628 | 0.1076 | No |
| 28 | YKT6 | na |  |  | 5213 | 0.597 | 0.1030 | No |
| 29 | PRPF3 | na |  |  | 5444 | 0.562 | 0.0975 | No |
| 30 | NPTXR | na |  |  | 5597 | 0.539 | 0.0959 | No |
| 31 | IRF1 | na |  |  | 5648 | 0.533 | 0.0995 | No |
| 32 | HTR7 | na |  |  | 5796 | 0.514 | 0.0978 | No |
| 33 | TYRO3 | na |  |  | 6080 | 0.475 | 0.0886 | No |
| 34 | GCH1 | na |  |  | 6157 | 0.463 | 0.0900 | No |
| 35 | FOS | na |  |  | 6167 | 0.461 | 0.0949 | No |
| 36 | MARK2 | na |  |  | 6269 | 0.447 | 0.0949 | No |
| 37 | HSPA2 | na |  |  | 6430 | 0.426 | 0.0915 | No |
| 38 | SQSTM1 | na |  |  | 7012 | 0.359 | 0.0653 | No |
| 39 | TGFBRAP1 | na |  |  | 7235 | 0.334 | 0.0575 | No |
| 40 | IL6 | na |  |  | 7244 | 0.333 | 0.0610 | No |
| 41 | RXRB | na |  |  | 7289 | 0.328 | 0.0626 | No |
| 42 | CXCL2 | na |  |  | 7437 | 0.314 | 0.0585 | No |
| 43 | RRAD | na |  |  | 7481 | 0.310 | 0.0599 | No |
| 44 | BTG2 | na |  |  | 7702 | 0.284 | 0.0517 | No |
| 45 | CDKN1C | na |  |  | 7826 | 0.269 | 0.0484 | No |
| 46 | POLR2H | na |  |  | 7921 | 0.259 | 0.0465 | No |
| 47 | H2AFX | na |  |  | 8328 | 0.216 | 0.0278 | No |
| 48 | BAK1 | na |  |  | 9101 | 0.141 | -0.0110 | No |
| 49 | PPIF | na |  |  | 9104 | 0.140 | -0.0095 | No |
| 50 | EPCAM | na |  |  | 9178 | 0.129 | -0.0118 | No |
| 51 | RHOB | na |  |  | 9250 | 0.124 | -0.0141 | No |
| 52 | CCNE1 | na |  |  | 9260 | 0.122 | -0.0131 | No |
| 53 | GLS | na |  |  | 9291 | 0.118 | -0.0133 | No |
| 54 | PPAT | na |  |  | 9363 | 0.109 | -0.0158 | No |
| 55 | AQP3 | na |  |  | 9407 | 0.104 | -0.0168 | No |
| 56 | ICAM1 | na |  |  | 9949 | 0.046 | -0.0446 | No |
| 57 | EPHX1 | na |  |  | 10016 | 0.038 | -0.0476 | No |
| 58 | TUBA4A | na |  |  | 10073 | 0.033 | -0.0502 | No |
| 59 | NAT1 | na |  |  | 10074 | 0.033 | -0.0498 | No |
| 60 | HLA-F | na |  |  | 10169 | 0.019 | -0.0545 | No |
| 61 | PRKCD | na |  |  | 10177 | 0.018 | -0.0547 | No |
| 62 | SPR | na |  |  | 10364 | 0.011 | -0.0643 | No |
| 63 | CNP | na |  |  | 10450 | 0.001 | -0.0687 | No |
| 64 | TFRC | na |  |  | 10516 | -0.007 | -0.0721 | No |
| 65 | HNRNPU | na |  |  | 10586 | -0.015 | -0.0755 | No |
| 66 | TAP1 | na |  |  | 10646 | -0.022 | -0.0783 | No |
| 67 | CHRNA5 | na |  |  | 10679 | -0.027 | -0.0797 | No |
| 68 | FEN1 | na |  |  | 10780 | -0.035 | -0.0845 | No |
| 69 | PDLIM3 | na |  |  | 10867 | -0.049 | -0.0885 | No |
| 70 | JUNB | na |  |  | 11042 | -0.071 | -0.0968 | No |
| 71 | PDAP1 | na |  |  | 11069 | -0.074 | -0.0973 | No |
| 72 | PRKACA | na |  |  | 11077 | -0.075 | -0.0967 | No |
| 73 | ENO2 | na |  |  | 11078 | -0.075 | -0.0959 | No |
| 74 | FKBP4 | na |  |  | 11149 | -0.086 | -0.0985 | No |
| 75 | CEBPG | na |  |  | 11400 | -0.101 | -0.1105 | No |
| 76 | DDX21 | na |  |  | 11428 | -0.105 | -0.1106 | No |
| 77 | CCND3 | na |  |  | 11490 | -0.109 | -0.1126 | No |
| 78 | EIF5 | na |  |  | 11523 | -0.115 | -0.1129 | No |
| 79 | SIGMAR1 | na |  |  | 11556 | -0.120 | -0.1132 | No |
| 80 | CYB5R1 | na |  |  | 11655 | -0.132 | -0.1167 | No |
| 81 | NFKBIA | na |  |  | 12331 | -0.218 | -0.1496 | No |
| 82 | GRINA | na |  |  | 12495 | -0.223 | -0.1555 | No |
| 83 | AP2S1 | na |  |  | 12760 | -0.254 | -0.1664 | No |
| 84 | TARS | na |  |  | 12761 | -0.254 | -0.1634 | No |
| 85 | C4BPB | na |  |  | 12944 | -0.277 | -0.1697 | No |
| 86 | PLCL1 | na |  |  | 13331 | -0.327 | -0.1861 | No |
| 87 | PSMC3 | na |  |  | 13373 | -0.334 | -0.1843 | No |
| 88 | DNAJB1 | na |  |  | 13393 | -0.336 | -0.1814 | No |
| 89 | SOD2 | na |  |  | 13651 | -0.378 | -0.1904 | No |
| 90 | LYN | na |  |  | 13668 | -0.380 | -0.1868 | No |
| 91 | HYAL2 | na |  |  | 13710 | -0.387 | -0.1844 | No |
| 92 | E2F5 | na |  |  | 13861 | -0.412 | -0.1874 | No |
| 93 | PARP2 | na |  |  | 14076 | -0.428 | -0.1936 | No |
| 94 | BSG | na |  |  | 14079 | -0.428 | -0.1887 | No |
| 95 | GRPEL1 | na |  |  | 14110 | -0.430 | -0.1852 | No |
| 96 | RFC4 | na |  |  | 14127 | -0.430 | -0.1810 | No |
| 97 | TST | na |  |  | 14230 | -0.448 | -0.1811 | No |
| 98 | BCL2L11 | na |  |  | 14467 | -0.486 | -0.1878 | No |
| 99 | CDC5L | na |  |  | 14582 | -0.503 | -0.1879 | No |
| 100 | GPX3 | na |  |  | 14790 | -0.536 | -0.1924 | No |
| 101 | PPP1R2 | na |  |  | 14880 | -0.545 | -0.1907 | No |
| 102 | ALDOA | na |  |  | 15303 | -0.618 | -0.2056 | Yes |
| 103 | HMOX1 | na |  |  | 15431 | -0.638 | -0.2047 | Yes |
| 104 | RPN1 | na |  |  | 15472 | -0.644 | -0.1993 | Yes |
| 105 | BID | na |  |  | 15537 | -0.659 | -0.1949 | Yes |
| 106 | POLE3 | na |  |  | 15652 | -0.677 | -0.1929 | Yes |
| 107 | BTG1 | na |  |  | 15833 | -0.711 | -0.1940 | Yes |
| 108 | MRPL23 | na |  |  | 15878 | -0.720 | -0.1879 | Yes |
| 109 | CYB5B | na |  |  | 15952 | -0.733 | -0.1831 | Yes |
| 110 | AMD1 | na |  |  | 16032 | -0.755 | -0.1783 | Yes |
| 111 | KLHDC3 | na |  |  | 16300 | -0.810 | -0.1828 | Yes |
| 112 | SLC25A4 | na |  |  | 16335 | -0.818 | -0.1750 | Yes |
| 113 | ALAS1 | na |  |  | 16364 | -0.825 | -0.1668 | Yes |
| 114 | EIF2S3 | na |  |  | 16381 | -0.828 | -0.1579 | Yes |
| 115 | TMBIM6 | na |  |  | 16758 | -0.916 | -0.1669 | Yes |
| 116 | STIP1 | na |  |  | 16929 | -0.964 | -0.1645 | Yes |
| 117 | BTG3 | na |  |  | 16989 | -0.983 | -0.1560 | Yes |
| 118 | CDK2 | na |  |  | 17319 | -1.090 | -0.1605 | Yes |
| 119 | ATP6V1F | na |  |  | 17588 | -1.191 | -0.1605 | Yes |
| 120 | ASNS | na |  |  | 18216 | -1.509 | -0.1757 | Yes |
| 121 | MAOA | na |  |  | 18217 | -1.509 | -0.1580 | Yes |
| 122 | CREG1 | na |  |  | 18238 | -1.522 | -0.1412 | Yes |
| 123 | UROD | na |  |  | 18338 | -1.593 | -0.1276 | Yes |
| 124 | RAB27A | na |  |  | 18362 | -1.614 | -0.1099 | Yes |
| 125 | HSPA13 | na |  |  | 18521 | -1.723 | -0.0980 | Yes |
| 126 | PPT1 | na |  |  | 18555 | -1.749 | -0.0791 | Yes |
| 127 | DNAJA1 | na |  |  | 18581 | -1.765 | -0.0597 | Yes |
| 128 | ATP6V1C1 | na |  |  | 18618 | -1.804 | -0.0404 | Yes |
| 129 | CASP3 | na |  |  | 18683 | -1.879 | -0.0217 | Yes |
| 130 | IL6ST | na |  |  | 18809 | -2.032 | -0.0044 | Yes |
| 131 | GGH | na |  |  | 18865 | -2.109 | 0.0175 | Yes |
Table: GSEA details [plain text format]

  

Fig 2: HALLMARK\_UV\_RESPONSE\_UP      
 Blue-Pink O' Gram in the Space of the Analyzed GeneSet

  

Fig 3: HALLMARK\_UV\_RESPONSE\_UP: Random ES distribution      
 Gene set null distribution of ES for **HALLMARK\_UV\_RESPONSE\_UP**
